# Reanalysis of EphA3 Knock-In Double Maps in Mouse Suggests That Stochasticity in Topographic Map Formation Acts at the Retina Rather than between Competing Mechanisms at the Colliculus

**DOI:** 10.1523/ENEURO.0135-23.2023

**Published:** 2023-11-16

**Authors:** David J. Willshaw, Nicholas M. Gale

**Affiliations:** 1 Institute for Adaptive and Neural Computation, School of Informatics, University of Edinburgh, Edinburgh EH8 9AB, United Kingdom; 2 Department of Applied Mathematics and Theoretical Physics, University of Cambridge, Cambridge CB3 0WA, United Kingdom

**Keywords:** double maps, EphA3 knock-in, Fourier-based imaging, mouse, superior colliculus, topographic map formation

## Abstract

It has been suggested that stochasticity acts in the formation of topographically ordered maps in the visual system through the opposing chemoaffinity and neural activity forces acting on the innervating nerve fibers being held in an unstable equilibrium. Evidence comes from the Islet2-EphA3 knock-in mouse, in which ∼50% of the retinal ganglion cells, distributed across the retina, acquire the EphA3 receptor, thus having an enhanced density of EphA which specifies retinotopic order along the rostrocaudal (RC) axis of the colliculus. Sampling EphA3 knock-in maps in heterozygotes at different positions along the mediolateral (ML) extent of the colliculus had found single 1D maps [as in wild types (WTs)], double maps (as in homozygous knock-ins) or both single and double maps. We constructed full 2D maps from the same mouse dataset. We found either single maps or maps where the visual field projects rostrally, with a part-projection more caudally to form a double map, the extent and location of this duplication varying considerably. Contrary to previous analyses, there was no strict demarcation between heterozygous and homozygous maps. These maps were replicated in a computational model where, as the level of EphA3 was increased, there was a smooth transition from single to double maps. Our results suggest that the diversity in these retinotopic maps has its origin in a variability over the retina in the effective amount of EphA3, such as through variability in gene expression or the proportion of EphA3+ retinal ganglion cells, rather than the result of competing mechanisms acting at the colliculus.

## Significance Statement

Analysis by others of visuocollicular maps in EphA3 knock-in mice indicated stochasticity in the development of nerve connections. Here, a proportion of the retinal ganglion cells have the chemoaffinity receptor EphA3. Sampling the heterozygous map had revealed either a single map [as in wild types (WTs)], a double map (as in homozygotes) or a mixture, suggesting an unstable balance between competing chemoaffinity and neural activity forces. We constructed full 2D maps from the same dataset. We found a diversity of double maps in both genotypes, with no demarcation between heterozygote and homozygote, replicated in a computational model where the level of EphA3 varies between animals. We suggest that any stochasticity acts at the level of the retina rather than downstream at the colliculus.

## Introduction

In mammals, retinal ganglion cells develop an ordered map of nerve connections across the superior colliculus (Dräger and Hubel, 1976; Finlay et al., 1978; King et al., 1996), driven by both chemical and electrical signaling (Pfeiffenberger et al., 2006; Cang and Feldheim, 2013). Chemical signaling involves the matching of axons carrying the EphA receptor, distributed as a monotonic gradient along the nasotemporal axis of the retina, with the collicular cells, carrying the ligand ephrinA, distributed as a monotonic gradient along the rostrocaudal (RC) axis. Correspondingly, the mapping of dorsoventral retina to mediolateral (ML) colliculus is mediated by gradients of EphB and ephrinB running dorsoventrally and mediolaterally, respectively (Flanagan and Vanderhaeghen, 1998; McLaughlin et al., 2003a).

In a wild-type (WT) map, there is a single ordered projection of the retinal nasotemporal axis onto the collicular rostrocaudal axis. In an EphA3 knock-in mouse, the EphA3 receptor, not normally present in mouse retina, is expressed by the Islet2 class of retinal ganglion cells which are distributed over the retina in equal proportions in a salt-and-pepper fashion (Brown et al., 2000). In the EphA3^ki/ki^ map, EphA3+ retinal cells project to rostral colliculus, with a similarly ordered projection from EphA3− cells more caudally, to form a double representation of visual field on colliculus. In the EphA3^ki/+^ map, the two types of cells also project separately except that the projections coincide in far rostral colliculus (Brown et al., 2000; Reber et al., 2004; Bevins et al., 2011). It was inferred that the degree of separation of the two projections on the colliculus depends on the extra amount of EphA assigned to EphA3+ cells, in the homozygous knock-ins being double that in the heterozygous knock-ins. (Brown et al., 2000; Reber et al., 2004).

**Figure 1.**
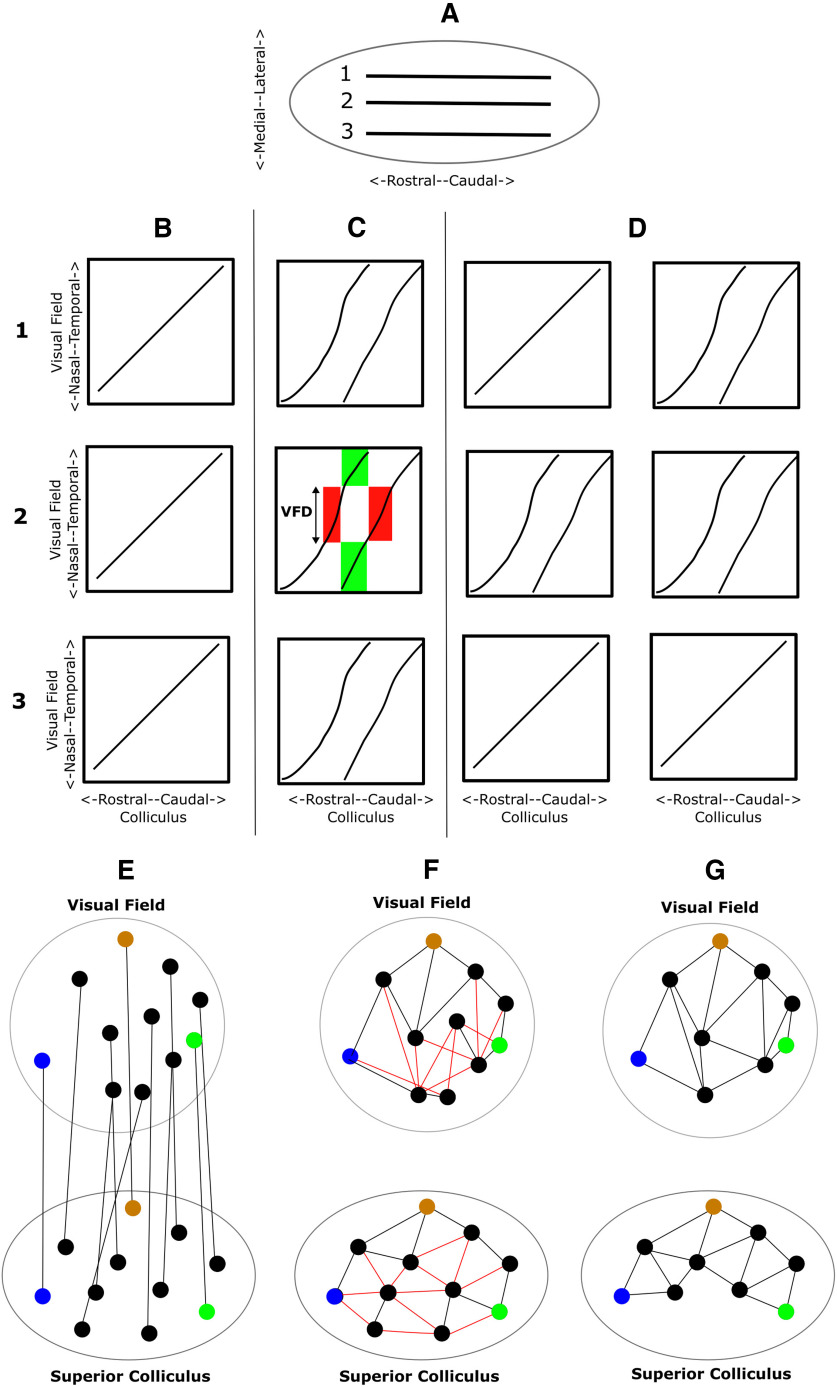
***A–D***, Schematic EphA3 knock-in visual maps of the 1D projection along the nasotemporal axis of visual field sampled along three different tracks 1, 2, 3 running rostrocaudally and spaced along the lateral-medial axis of the colliculus, used to illustrate the findings of Owens et al. (2015). These maps are also used to show the general properties of EphA3 knock-in maps in 1D. The shapes of the 1D profiles were redrawn from Brown et al. (2000). ***A***, Superior colliculus showing the three tracks. ***B***, Wild type. Mechanisms of neural activity and chemoaffinity combine to form a single projection along all three tracks. ***C***, Homozygous knock-in (EphA3^ki/ki^). Double maps along all three tracks. With high EphA3 in the Islet2 retinal ganglion cells, the EphA3+ and the EphA3− retinal ganglion cells each make a separate map. Green areas in panel 2 enclose the connections made from two distinct parts of the visual field to the same part of the colliculus. Red areas enclose the connections made from the same part of the visual field to two distinct regions of the colliculus, with its extent measured by the amount of visual field duplication (VFD). ***D***, Heterozygous knock-in (EphA3^ki/+^). In addition to entirely single and entirely double maps, in some cases there is a mixture of these two types. Two possible explanations are the collicular hypothesis and the retinal hypothesis. The collicular hypothesis: with a low level of EphA3 in the heterozygote, the mechanisms of neural activity and chemoaffinity on the colliculus are finely opposed in an unstable equilibrium so that all combinations of maps may be seen along the three tracks (Owens et al., 2015). The retinal hypothesis: since double maps are associated with high EphA3 and single maps with zero EphA3, there is a variation of the level of EphA3 across the retina leading to different types of map along different tracks on the colliculus. ***E–G***, Use of the Lattice Method to construct an ordered one-to-one 2D map. ***E***, Virtual electrodes are placed on the colliculus to cover the surface. They are added one by one at random positions subject to the constraint that each new electrode is at a prespecified distance Δ (±10%) from all existing electrodes. The procedure terminates once no more suitable positions can be found. For each electrode the corresponding position in the visual field is then found by averaging over all visual field positions associated with the points on the colliculus within a distance Δ/2 of the given electrode position. ***F***, Nearest neighboring electrodes on the colliculus are interconnected using Delaunay triangulation to form a lattice. Each pair of visual field points (nodes) where the corresponding electrodes are nearest neighbors are also interconnected. If the map has perfect order, the collicular lattice will be replicated on the visual field. If this is not the case, links in the visual field will cross over showing that neighborhood relations are violated. These links are colored red and the corresponding links on the collicular lattice are also colored red. ***G***, Pairs of electrodes and the corresponding visual field nodes are removed one by one to eliminate the cross-overs from the visual field representation. Ultimately, the visual field representation becomes a lattice, indicating a perfectly ordered but reduced map. The elimination method yields a lattice containing no more than 8% of the optimal number of nodes found by linear programming where different part of the map may be disconnected (Willshaw et al., 2014). Three pairs of matching colored points in the two structures indicate the global orientation of the map. In this schematic example, two of the eleven points had to be removed, showing that the ordered map is restricted to one part of the colliculus. Various metrics can be derived from these plots, such as the percentage number of nodes in the perfectly ordered lattice compared with the original as a measure of Map Quality, and the polarity and the magnification of the map along any specified axis.

Owens et al. (2015) explored the maps in EphA3^ki/+^ animals using Fourier-based intrinsic imaging, where the visual field is scanned over two orthogonal directions. (Kalatsky and Stryker, 2003). They sampled the azimuthal scan to construct the 1D nasotemporal to rostrocaudal projection at three different dorsoventral locations. They found either single, double or a mixture of single and double maps. They proposed that this variety among EphA3^ki/+^ maps is evidence for stochasticity operating in the formation of nerve connections: that in the EphA3^ki/+^ animal, activity-based forces favoring single maps are pitted against chemically-based forces favoring double maps in an unstable equilibrium, small fluctuations resulting in either a single or double map or a mixture (Fig. 1*A–D*).

Since the level of EphA3 assigned to Islet2 retinal ganglion cells determines whether a double or single map results, another explanation of these results is that the level of EphA3 in EphA3^ki/+^ animals varies over the retina. To distinguish both hypotheses (legend to Fig. 1) requires information about the entire map from both directions of scan. We analyzed the same dataset. Following Cang et al. (2008), we excluded noisy data, and also data from collicular regions where the map is discontinuous, suggesting two or more visual areas mapping to the same collicular area. These cannot be discriminated since the imaging method gives a single point in the visual field for each pixel in the collicular map. We then reconstructed the 2D visuocollicular map employing the Lattice Method (Willshaw et al., 2014), finding either single maps or maps in which the visual field projected rostrally and caudally to form a double map, the extent and location of the caudal part varying considerably; there was no strict demarcation between EphA3^ki/+^ and EphA3^ki/ki^ maps. In these maps there is no information about how the two populations of EphA3− and EphA3+ retinal ganglion cells project onto the colliculus. Therefore, to help examine possible modes of projection we used simulation results from computer models (Triplett et al., 2011; Hjorth et al., 2015) where such information is available. We found no evidence for stochasticity operating at the colliculus but instead our results suggest that upstream variabilities in EphA3 signaling may be responsible.

## Materials and Methods

We analyzed Fourier-based intrinsic imaging data from EphA3 knock-in mice of either sex, originally mapped by Owens et al. (2015).

### Filtering the imaging data

In Fourier-based intrinsic imaging, the visual field is scanned in two orthogonal directions to determine separately the mapping of visual field positions along the nasotemporal axis onto the colliculus (azimuthal scan) and that of positions along the dorsoventral axis of visual field (elevational scan). Following Kalatsky and Stryker (2003), we defined the location of the visual projection on the colliculus as the region where excitation from visual field in the elevational map (dorsoventral scan) was highest, and an ellipse was drawn to enclose this region. Here, the strength of the signal varied considerably and information from only the 10,000 most active pixels was considered (Cang et al., 2008). By combining data from the azimuthal and elevational scans of the visual field, the visual field position providing maximal excitation at each pixel in the collicular image was then calculated (Willshaw et al., 2014).

A second stage of filtering was then necessary to remove unreliable information resulting from use of the Fourier imaging method. As shown for 1D maps in Figure 1, this method yields a single position in the visual field for each position on the colliculus. This is appropriate for a normal, single projection (Fig. 1*B*), or for the part of a double map where there is an ordered map to two separate areas of the colliculus (shown for a 1D map by the red areas in Fig. 1*C*). In these cases, a unique position in the visual field can be identified with each point on the colliculus. The visual field position identified with each small area of colliculus varies smoothly over the colliculus. However, in cases where two small regions of visual field map to the same area of colliculus (Fig. 1*C*, green area), this average visual field position recorded at any pixel will be very different from the two visual field positions and therefore inaccurate. This average is liable to fluctuate of pixel to pixel (Willshaw et al., 2014) and therefore pixels for which the standard deviation of the mean fluctuation was greater than three times than that in WTs were removed from the analysis.

The active pixels remaining after these two stages of filtering are called eligible and those discarded are called rejected. Visual field information derived from the set of eligible pixels was then used to construct 1D and 2D maps.

### Extension of 1D analysis

To establish a baseline for comparing directly with the method of Owens et al. (2015), we plotted out the variation of azimuthal phase, signaling the nasotemporal component of visual field, along three straight lines drawn on the colliculus, which was intended to represent the visual field at the dorsoventral elevations of −25°, 0°, and +25°. We found the projection onto the colliculus from all visual field positions at the given elevation and then made a straight line fit to these points. This line was then projected back onto the visual field and the corresponding visual field positions plotted.

### Construction of 2D maps, the Lattice Method

We used the Lattice Method (Willshaw et al., 2014), developed to analyze another set of Fourier-based intrinsic imaging data of the mouse visuocollicular projection (Cang et al., 2008). Eligible pixels were chosen at random to be “virtual electrodes”, subject to there being an approximate spacing of 6 pixel-widths (here equivalent to 80 μm) between nearest neighbors. For each virtual electrode, we then calculated the mean position of all visual field points having corresponding collicular positions within three pixel-widths from the virtual electrode. This gave a one-to-one mapping between 150–200 virtual electrodes on the colliculus (collicular nodes), spaced at ∼80 μm, and the same number of averaged visual field positions (visual nodes). The spacing between collicular nodes was chosen to be the smallest that gives highly ordered 2D maps in WTs, as found in the previous analysis (Willshaw et al., 2014). Figure 1*E* shows a simple example of a one-to-one mapping.

Neighboring collicular nodes were then interconnected to form a lattice. The nodes in the visual field which are associated with interconnected collicular nodes were also interconnected (Fig. 1*F*). If the resulting structure constructed in the visual field is also a lattice, with no edges crossing, then local order is preserved because each pair of neighboring visual nodes then connects to a pair of neighboring collicular nodes. Otherwise, removal of selected pairs of matching visual and collicular nodes yields the largest ordered submap in which the remaining visual nodes form a lattice (Fig. 1*G*). Map Quality, the proportion of nodes remaining in the largest ordered submap, is a measure of the degree of local order.

In cases where two projection areas on the colliculus were defined by the distribution of eligible pixels, two separate partmaps were also constructed from the two lattices made from the nodes in the corresponding areas. This removed the interference caused by portions of the visual field being represented in both areas of the colliculus (Fig. 1*C*, green areas).

Global properties of the map were measured in the largest ordered submap, by comparing the properties of the lattice and the corresponding visual field lattice so formed. They are assessed along two orthogonal axes after rotating the projection in visual space by 20° to allow for the fact that in WT maps the nasotemporal axis projects at this angle onto the rostrocaudal axis (Dräger and Hubel, 1976; Willshaw et al., 2014).

Order along selected axes of the map was measured. Rostrocaudal polarity is the proportion of edges in the visual lattice which project onto the colliculus in the same relative rostrocaudal order as they would in a perfectly ordered map. A score of 100% represents perfect ordering along the rostrocaudal axis, 50% indicates random order and perfect but reversed order is 0%. Mediolateral polarity is calculated similarly.

Magnification ratio is a measure of the relative extent of the projection along a specified axis. The azimuthal magnification (AM) is the length of each edge in the visual lattice measured along the rotated nasotemporal axis of visual field compared with the length of the corresponding edge in the collicular lattice measured along the rostrocaudal axis; similarly for the elevational magnification (EM).

In cases when two partmaps were constructed by partitioning the colliculus, the extent of visual field represented in both regions of the colliculus, here called the visual field duplication (VFD), was measured. In a completely double map, the VFD is the entire extent of visual field. In Figure 1*C*, there is a partial double map and the extent of the 1D VFD is shown (red area).

### Computer simulations

A computational model (Triplett et al., 2011; Hjorth et al., 2015) for the development of a retinocollicular map, here called TK2011, was used to help to understand the data.

In this model, retina and colliculus are each laid out as a 2D array of cells. The mapping is built up by iteratively creating and deleting contacts to reduce a quantity called the energy, calculated over all contacts, which reflects the influences of chemoaffinity, neural activity and competition. Each cell may have more than one contact.

Each newly created contact is chosen by sampling at random from all possible pairs of cells. Similarly, each possible deletion is chosen from the existing set of contacts. The chemoaffinity component of the energy is of the graded matching (Prestige and Willshaw, 1975) rather than the direct matching type (Sperry, 1943, 1963). It is calculated from the gradients of EphA and EphB labeling the two axes of the retina and the matching gradients of ephrinA and ephrin B in the colliculus. For a retinal cell with an amount R_A_ of EphA and a collicular cell with an amount C_A_ of ephrinA, the contribution to the energy for the contact between them is the product R_A_xC_A_. This favors contacts between cells with high EphA and those with low ephrinA, and vice versa, which replicates the directions of the EphA and ephrinA gradients found experimentally (Cheng et al., 1995; Flanagan and Vanderhaeghen, 1998). In the EphB/ephrinB system, high EphB maps to high ephrinB (McLaughlin et al., 2003a) and so the contribution to the energy is −R_B_xC_B._ The contribution to the energy from electrical activity favors the development of contacts from neighborhoods to neighborhoods and the competitive mechanism favors the creation of synapses in high-density locations and their deletion in low-density locations.

We simulated the development of the retinocollicular map using TK2011 with the code developed by Hjorth et al. (2015). To match to the number of data points selected for analysis of the experimental data, 10,000 retinal cells were distributed randomly within a circular retina subject to a minimal distance between neighbors. Ten thousand collicular cells were distributed in the same way, over a hemi-ellipse representing the shape of the colliculus. The shapes of the EphA profiles were taken from Reber et al. (2004), with values scaled so that in the wild type they were between 0 and 1. We used the parameter values from Triplett et al. (2011) except that the magnitude of the activity term in the energy was scaled to ensure that in the wild-type virtual injections of 1% of the retinal area innervated 1% of the colliculus (McLaughlin et al., 2003b) and we ran each simulation for a larger number of iterations, 5 × 10^8^ against 1.4 × 10^7.^ The knock-in of EphA3 was modelled by adding an extra amount of EphA, DR, to ∼50% of the population of retinal cells selected randomly. Choosing a roughly equal number of cells to be EphA3+ and EphA3− removes any competitive effect because of different numbers of cells. For EphA3^ki/ki^ animals the value of DR was assumed to be twice that for EphA3^ki/+^ animals (Brown et al., 2000). To investigate any variation because of the random positioning of cells, the random assignment of retinal cells to be EphA3+ and the randomness of the addition and deletion of synapses, a number of runs on a reduced 2000 × 2000 system was also conducted for each value of DR.

To model the experimental results precisely we then made a functional map by taking the modelled anatomic data obtained from the initial simulations and then replicating the Fourier imaging method. We simulated the repeated scanning of the retina in two orthogonal directions using a bar with a width of 2% of the linear extent of the retina (Kalatsky and Stryker, 2003). Retinal and collicular firing rates were calculated using a Poisson model, with retinal firing rate proportional to the location of the bar and collicular firing rate calculated from the transmission of retinal spikes through the contacts formed and the transmission of collicular spikes through local excitation and inhibition (Phongphanphanee et al., 2014). In order to extract the mapping of collicular position onto retinal position, we then conducted Fourier analysis of the time course of the activity pattern at the repetition frequency. We constructed the map from colliculus to retina, which corresponds to the experimental maps found by Fourier intrinsic imaging. We also constructed simulated maps to the colliculus from the whole retina and from the separate subpopulations of EphA3+ and EphA3− retinal ganglion cells. In the latter two cases, the scan was restricted to the subpopulation in question. All 2D maps were constructed using the Lattice Method (Willshaw et al., 2014).

To compare with the modeling results obtained by Owens et al. (2015), we also simulated an earlier version (Tsigankov and Koulakov, 2006, 2010) of this model, here called TK2006, The crucial difference between the two models is that in TK2006, each collicular cell makes a single contact. This removes the need for a competition term in the energy function but at the expense of introducing discontinuities in the mapping.

### Access to resources

For access to the experimental data, please refer to Owens et al. (2015), where the data were first reported. The simulation code for TK2011 developed by Hjorth et al. (2015) is available at https://github.com/Hjorthmedh/RetinalMap. The code used for the analysis of the experimental and the simulation data is under development and the current version is at https://github.com/Nick-Gale/Lattice_Method_Analysis_EphA3_Mutants.

## Results

The dataset is from 24 mice, five wild types (WTs), five homozygous EphA3 knock-ins (HOM) and 16 heterozygous EphA3 knock-ins (HET), previously analyzed by Owens et al. (2015). We could not analyze the data which these authors used to explore the roles of neural activity and genetic variability owing to there being no matching azimuthal and elevational scans in the database.

### Distributions of eligible pixels

**Figure 2.**
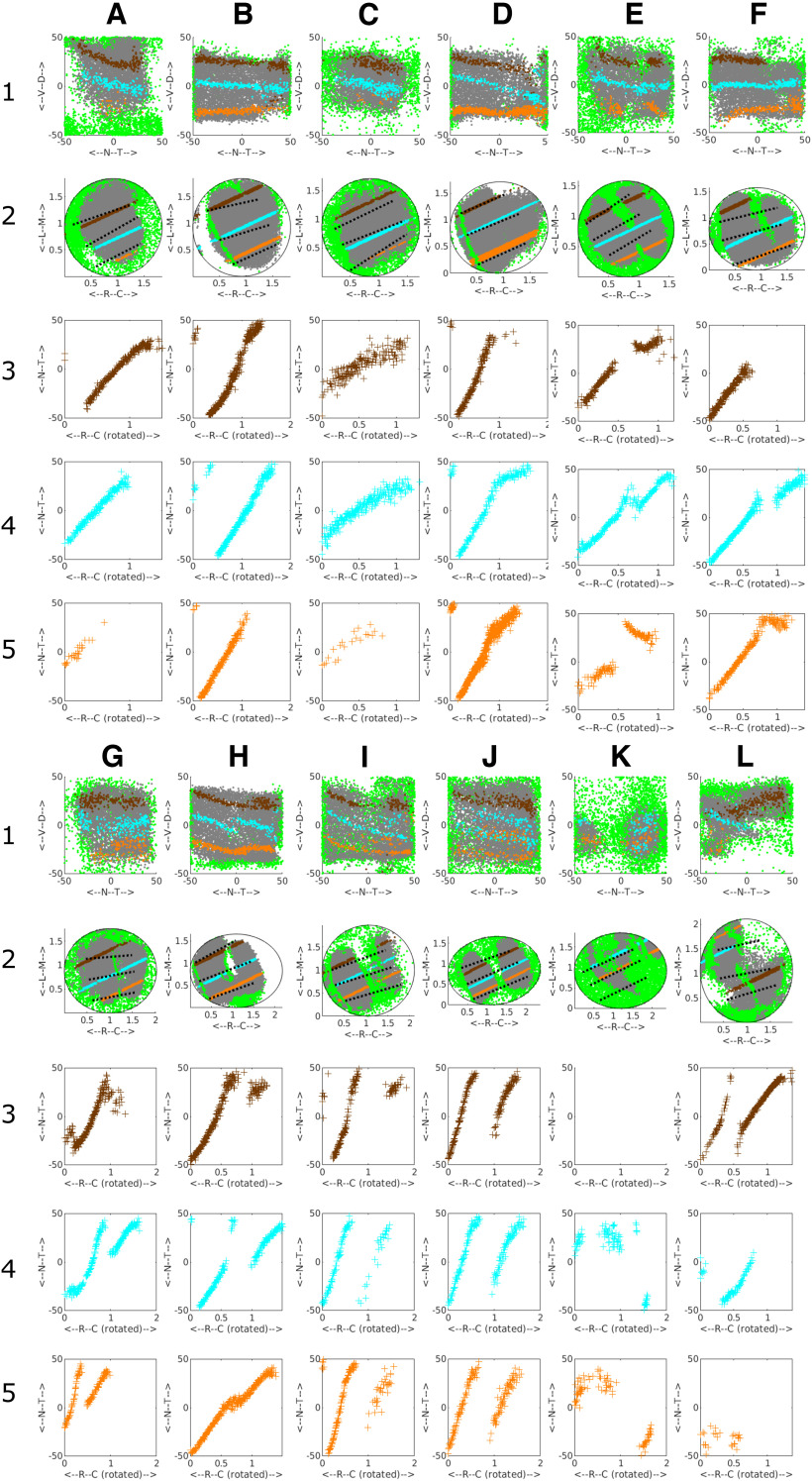
Examples from the database of the distribution of the variation of the azimuthal (nasotemporal) component of phase representing visual field position recorded along three straight line tracks drawn on the colliculus representing 1D sections at three standard elevations of −25° (orange), 0° (cyan), and +25° (brown) for WT, heterozygous (EphA3^ki/+^) and homozygous (EphA3^ki/ki^) knock-ins. ***A***, ***B***, WT. ***C–H***, ***K***, EphA3^ki/+^. ***I***, ***J***, ***L***, EphA3^ki/ki^. Rows 1, 2 show the distributions of eligible (gray) and rejected (green) pixels on the colliculus (row 2) and corresponding visual field positions (row 1). Each colored line on the colliculus (row 2) is the straight line fit to the positions of all eligible pixels for which the corresponding visual field positions are at the given elevation. The colored points plotted on the visual field representation is the projection back into the visual field from all the pixels on the given straight line. These projections are not aligned perfectly with the appropriate elevations, suggesting that the nasotemporal and dorsoventral aspects of the map cannot be separated easily. Black dotted lines on the colliculus indicate the tracks used by Owens et al. (2015) in sampling the projection. Rows 3–5, Plots of the azimuthal component of phase against collicular position along the appropriate track. The orientation of the 1D sections vary but all are seen to be at ∼20° to the rostro-caudal axis. All three plots for the same dataset are drawn with the same horizontal scale, which varies from dataset to dataset. No data were recorded in the plot in ***K***, row 3. Units of phase are degrees and those of position on the colliculus are mm.

Figure 2 shows twelve examples of distributions of the gray (eligible) pixels used to construct the map after filtering to reject the green pixels.

In WTs (Fig. 2*A*,*B*) there is one large area of gray eligible pixels on the colliculus (row 2), taken to be the main projection area, surrounded by rejected pixels. In Figure 2*B*, there is a small and thin secondary projection area, seen also in Figure 2*D*. Heterozygous EphA3 knock-ins (HET; Fig. 2*C–H*) show a variety of projection areas. In some cases, there is one main area of eligible pixels (Fig. 2*C*,*D*); in others, there are signs of two substantial projection areas with rejected pixels between them of various forms (Fig. 2*E–H*). Homozygous EphA3 knock-ins (HOM), show a clear separation of the colliculus into two projection areas (Fig. 2*I*,*J*). Figure 2*K*,*L* shows examples of the three cases which did not show well-defined projection areas and so were not analyzed further.

### 1D analysis: sampling the nasotemporal to rostrocaudal projection

To motivate our 2D analysis and compare our 1D results with that of Owens et al. (2015), in Figure 2 we also show the 1D projections at three standard elevations in the visual field (rows 3–5). The projections are from colliculus (row 2) to visual field (row 1). Most of the 21 cases analyzed extensively each had a strong 1D map, extending from nasal field to cover most of the visual field (rows 3–5). In some of the profiles the map was absent or did not cover all of visual field (Fig. 2*A*,*C*, row 5, *F*, row 3), or had breaks (Fig. 2*E*, rows 3, 5, *F*,*H*, row 4) In both the HOMs and the HETs, there was also a second projection from caudal colliculus of a variable size, which was restricted to temporal field (Fig. 2*G–J*, rows 3–5, except *H*, row 5). There were also other types of projection such as the small projection to temporal field from rostral colliculus (Fig. 2*B*,*D*, rows 3–5).

In summary, the 1D projections are highly variable. Viewing how the tracks along which the 1D profiles were taken run over the irregular regions of eligible pixels on the colliculus (row 2) suggests that the 1D profiles reconstructed are sensitive to the exact positioning of the tracks. We conclude that assessing the projection at three standard positions does not allow for the variability in the maps and the nature of the projections is more complex than a binary classification into single or double projections (Owens et al., 2015). This gave additional motivation to pursue the construction of 2D maps.

### Wild-type 2D maps

To establish a baseline, the five WTs in the dataset were analyzed. In each case there is a single large and extremely well-ordered projection, including two with a small aberrant projection.

**Figure 3.**
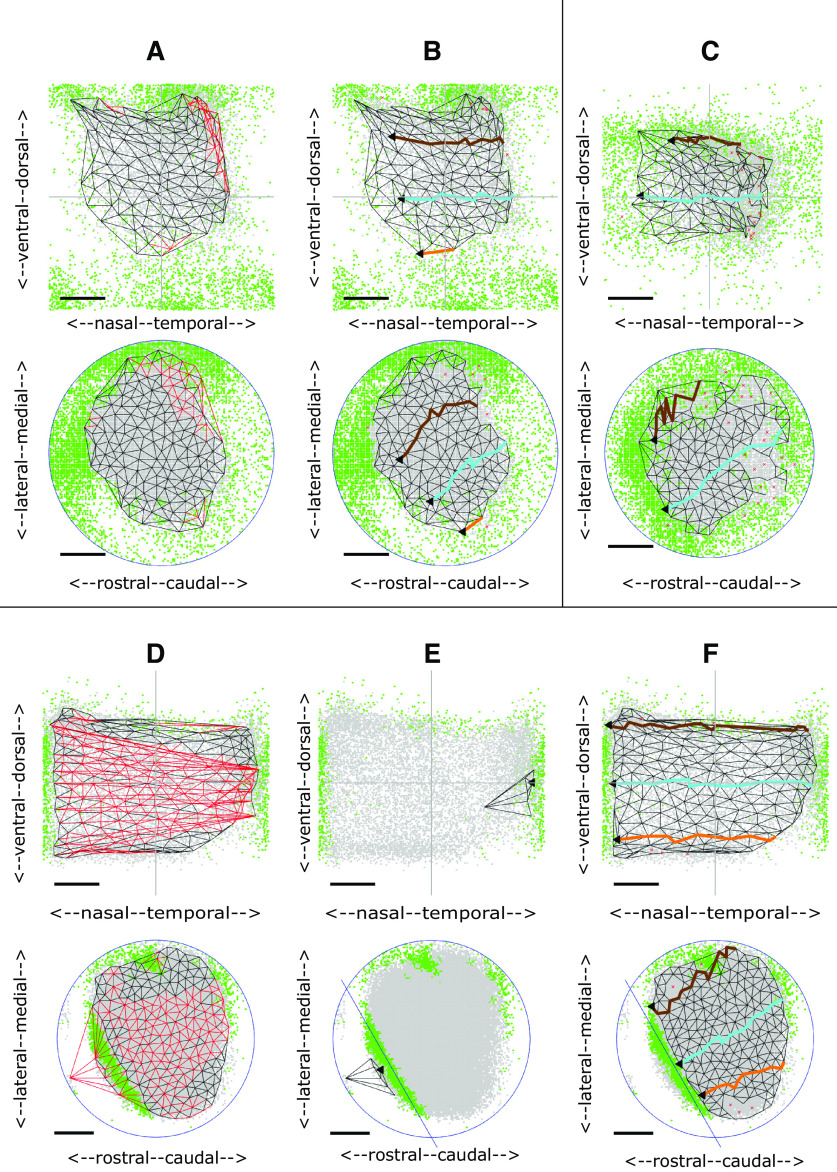
2D maps from colliculus (bottom) to visual field (top) for WT and HETA datasets. Neighboring nodes on the colliculus and the corresponding nodes in the visual field map are connected by straight lines. Lines in the visual field which cross and the corresponding lines on the colliculus are colored red. To relate the 2D maps to the patterns of 1D profiles in Figure 2, in some figures three lines representing the projections at −25° (orange), 0° (cyan),^ ^and +25° (brown) are shown. ***A***, ***B***, Maps formed for the WT shown in Figure 2*A*. ***A***, The complete projection with 162 nodes. ***B***, The largest ordered submap, constructed by removing 4% of the nodes from the complete projection to eliminate all crossing lines giving a Map Quality of 96%. RC, ML polarities: all 90%. ***C***, The largest ordered submap for the HETA shown in Figure 2*C*, with 215 nodes in the whole map and 183 in the largest ordered submap giving a Map Quality of 86%. RC, ML polarities: all 90%. Azimuthal and elevational magnifications: 77°/mm, 60°/mm. ***D–F***, Maps formed for the WT shown in Figure 2*B*. ***D***, The complete projection, with 165 nodes. ***E***, ***F***, The two largest ordered subpartmaps constructed on the two sets of nodes by partitioning the set of collicular nodes with a line drawn through the narrow strip of rejected pixels in rostral colliculus. ***E***, Subpartmap on rostral colliculus. ***F***, Subpartmap on the rest of the colliculus. RC, ML polarities are 94%. AM, EM: 115°/mm, 58°/mm. Scale bars: 20° (visual field); 200 μm (colliculus).

Figure 3*A* shows the complete projection between each of the nodes on the colliculus (bottom) to the matching nodes in the visual field (top) for the WT shown in Figure 2*A*. The red links, mainly in temporodorsal visual field and caudomedial colliculus, show regions where links in the visual field cross, indicating violation of neighborhood relations. Removal of selected nodes caused these links to be deleted until the largest ordered submap with perfect local order remained (Fig. 3*B*). To give a sense of the overall polarity of the map and to relate these maps to the 1D slices shown in Figure 2*A*, three lines running nasotemporally which link the visual field nodes closest to the same standard elevations used in the 1D analysis were projected onto the colliculus. The map in Figure 3*B* has high local order with Map Quality, the percentage number of nodes in the largest ordered submap, of 96%. The rostrocaudal (RC) and mediolateral (ML) polarities, defined in Materials and Methods, are in excess of 90%.

Figure 3*D–F* show another WT map with a strip of rejected pixels separating a large area of eligible pixels from a small area (Fig. 2*B*). In the whole map (Fig. 3*D*) there are many violations of neighbor relations. By dividing the collicular nodes into two by a line drawn through the strip of green rejected pixels and then constructing separate maps on the two collicular lattices so formed (Fig. 3*E*,*F*), the small projection area rostrally was found to be associated with temporal field (Fig. 3*E*). The massive violation of neighbor relationships seen in Figure 3*D* is because of this small part of temporal field being represented both rostrally and caudally.

In the group of five WTs, the number of nodes on the colliculus is 188 ± 38, spanning an ∼1 mm^2^ circular region. With a Map Quality of 91 ± 4%, around 90% of the nodes are positioned in the correct relative order at an internode spacing of 80 ± 8μm in the colliculus. The RC and ML polarities are 90 ± 4% and 91 ± 3%. The map is slightly more elongated along the (rotated) rostrocaudal axis than the (rotated) mediolateral axis. Azimuthal magnification (AM) and elevational magnification (EM) are 93 ± 13°/mm, 71 ± 9°/mm.

### EphA3^ki/+^ 2D maps (HETS)

Two datasets had been discarded at the 1D analysis stage, one of these shown in Figure 2*K*. The remaining 14 cases fall into three groups.

#### HETA (five cases)

In this group there is one projection area on the colliculus and a single ordered map. The largest ordered submap of one example is shown in Figure 3*C*, with pixel distributions in Figure 2*C*. These five maps are slightly less well-ordered than WTs, with Map Quality 86 ± 2% against 91 ± 4%. The number of nodes (190 ± 22 against 188 ± 38), the RC and ML polarity scores (91 ± 2%, 90 ± 1% against 91 ± 4%, 91 ± 3%) and the magnifications (98 ± 16°/mm, 63 ± 5°/mm against 93 ± 13°/mm, 71 ± 9°/mm) are very similar to those in WTs (Fig. 9*A–F*).

In three cases, a small part of nasal field is associated with far caudal colliculus. One example of the associated pixel distribution is shown in Figure 2*D*. The maps are similar to those found in two WTs (Fig. 3*D–F*). These suggest a slight phase miscalibration in the scanning, demonstrating the use of the Lattice Method to disentangle ordered mappings.

#### HETB (four cases)

**Figure 4.**
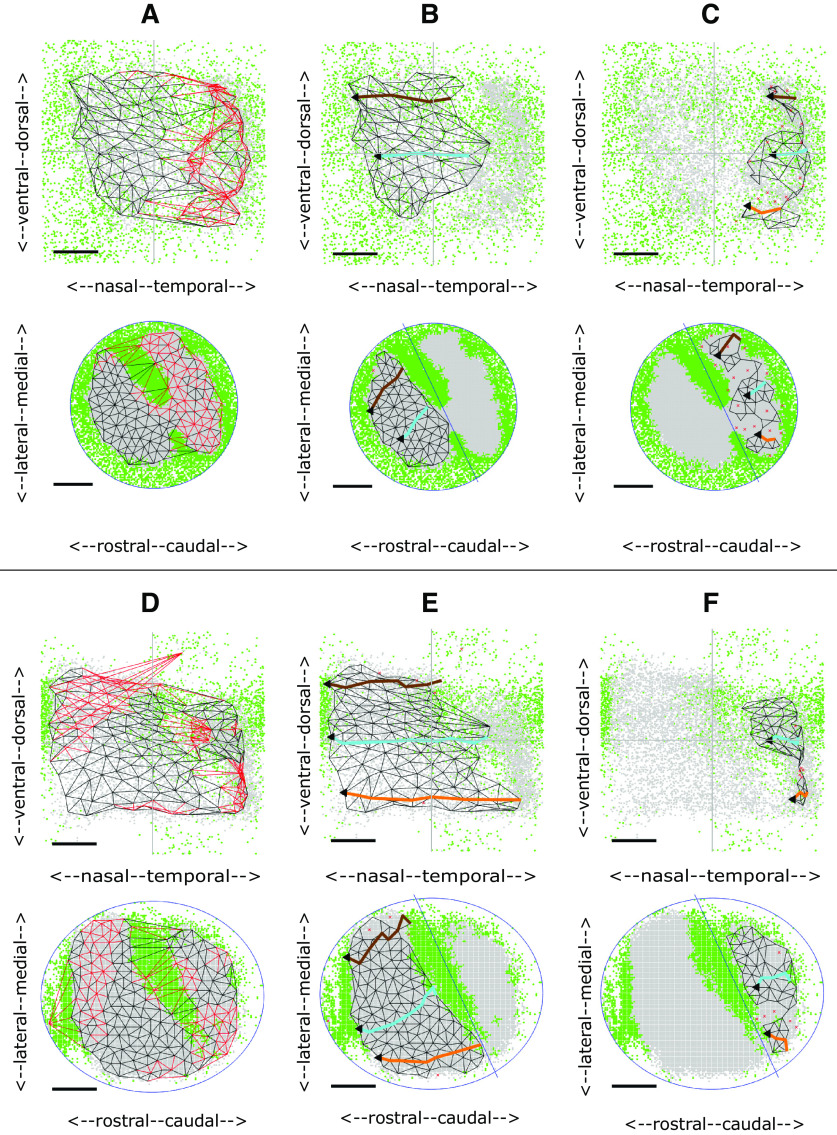
2D maps for two HETB datasets. **A–C.** Maps formed from the data shown in Figure 2*E*. ***A***, Whole map with 148 nodes. Largest ordered submap has 124 nodes with Map Quality 84%. ***B***, ***C***, Subpartmaps formed after partitioning the colliculus through the green areas of rejected pixels into rostral (***B***) and caudal (***C***) areas; 3% more nodes were recruited in the two subpartmaps than in the largest ordered submap, giving a Map Quality of 87%. VFD, the area of visual field contained in both rostral and caudal projections: 3%. RC, ML polarities and AM, EM: 94%, 89%, 118°/mm, 89°/mm (***B***); 68%, 83%, 84°/mm, 75°/mm (***C***). ***D–F***, Maps formed from the data shown in Figure 2*F*. ***D***, Whole map with 186 nodes. The largest ordered submap has 169 nodes giving a Map Quality of 91%. ***E***, ***F***, Rostral and caudal subpartmaps, with 3% more nodes than in the largest ordered submap. VFD: 7%. RC, ML polarities and AM, EM: 96%, 92%, 101°/mm, 92°/mm (***E***); 68%, 92%, 86°/mm, 62°/mm (***F***). Conventions as in Figure 3.

The two regions of eligible (gray) pixels on the colliculus suggest two incompletely separated projection areas with rejected (green) pixels interposed (Fig. 2*E*,*F*). These could represent noise or regions associated with two distinct areas of visual field and thus cannot be mapped. Figure 4 shows the maps for these two HETs. Here, the rostral and caudal projection areas were defined by drawing a line through the narrow neck of the composite region to produce separate largest ordered subpartmaps (Fig. 4*B*,*C*,*E*,*F*). Map Quality computed over the two subpartmaps is 3% more than in the single map.

In this category, the two separate partmaps make up a single map with only a very small area of visual field represented on both rostral and caudal subdivisions of the colliculus, the extent measured by the visual field duplication (VFD). The area of visual field associated with rostral colliculus is 74 ± 17% of the total occupied by the largest ordered submap against 30 ± 15% to caudal colliculus, giving a VFD of 4 ± 3%. Defining the total collicular region as the areas occupied by the rostral and caudal regions together with the area between them, the rostral region occupies 48 ± 13% of the colliculus, the caudal region 33 ± 10% and the central region 19 ± 10%.

Map Quality of the two partmaps is 93 ± 4% compared with 91 ± 5% for the whole map, identical to the figure for WTs. The RC and ML polarities in both the largest ordered single subpartmap and the largest ordered subpartmap in rostral colliculus are all in excess of 90%, at the WT level, whereas for the smaller projection to caudal colliculus the polarities are slightly lower (Fig. 9*A–D*).

#### HETC (five cases)

**Figure 5.**
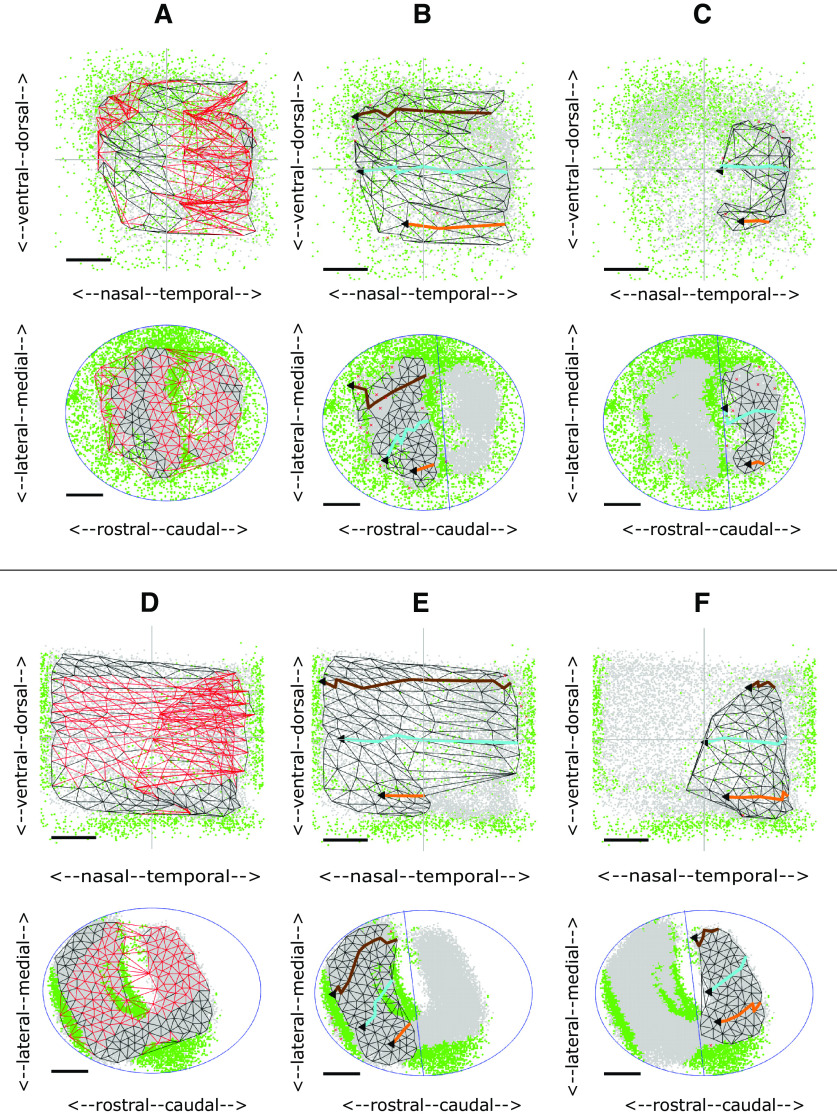
2D maps for two HETC datasets. ***A–C***, Maps formed from the data shown in Figure 2*G*. ***A***, Whole map with 180 nodes in the complete map. Largest ordered submap has 135 nodes with Map Quality 75%. ***B***, ***C***, Subpartmaps, with Map Quality of 85%, 10% more than in the largest ordered submap. VFD: 29%. RC, ML polarities and AM, EM: 91%, 85%, 168°/mm, 84°/mm (***B***); 90%, 88%, 88°/mm, 73°/mm (***C***). The value of AM for ***B*** is almost twice that for ***C***, primarily because of the high magnification in temporal field. ***D–F***, Maps formed from the data shown in Figure 2*H*. ***D***, Whole map with 207 nodes. Largest ordered submap has 188 nodes with Map Quality 91%. ***E***, ***F***, Subpartmaps, with Map Quality 98%, 7% more than in the largest ordered submap. VFD: 29%. All polarities between 90% and 93%. AM, EM: 160°/mm, 82°/mm (***E***); 86°/mm, 76°/mm (***F***). Conventions as in Figure 3.

Here, there are also suggestions of two projection areas (Fig. 2*G*,*H*). In this group, two well-ordered partmaps could be identified with polarity scores and Map Quality greater than for the whole map (Fig. 9*A*,*C*,*D*); 91 ± 11% of the visual field is represented in rostral colliculus and 27 ± 14% in caudal colliculus giving larger VFDs than for HETBs, of 18 ± 7%. Two HETCs, corresponding to Figure 2*G*,*H*, are shown in Figure 5. In both cases, whereas the projection from the rostral area is to the entire visual field (Fig. 5*B*,*E*), there is no projection to nasal visual field from the caudal area (Fig. 5*C*,*F*). This results in a higher AM than EM and significant VFD of 27% and 24%, respectively (Fig. 9*E*,*F,B*). The averages sizes of the rostral region of projection, the caudal region and the central region are similar.

### EphA3^ki/ki^ (HOM) 2D maps

Four of the five available datasets were analyzed, the fifth being rejected as a result of the 1D analysis (Fig. 2*L*). In these four HOMs, partitioning the colliculus into two yields two distinct, well ordered partmaps. They have high polarity scores and Map Quality is 89 ± 10%, substantially greater than 71 ± 10% for the whole projection (Fig. 9*A*,*C*,*D*). The rostral projection area has a projection to almost all (98 ± 4%) of the visual field whereas the caudal region has a projection to about one third (35 ± 20%), in temporal field. As a result, the VFD is substantial, varying widely with individual scores of 17%, 25%, 29%, and 59% of the visual field, an average of 33 ± 18*%.* The sizes of the rostral projection area, the caudal projection area and the area between them are very similar (32 ± 10%, 32 ± 13%, 35 ± 7%). The fact that a larger area of the visual field is associated with the rostral area than with the caudal area of the same size is reflected in the fact that the azimuthal magnification (AM) for the rostral partmap is double that for the caudal map: 203 ± 20°/mm against 101 ± 16°/mm, which is similar to the WT value of 93 ± 13°/mm (Fig. 9*E*,*F*).

**Figure 6.**
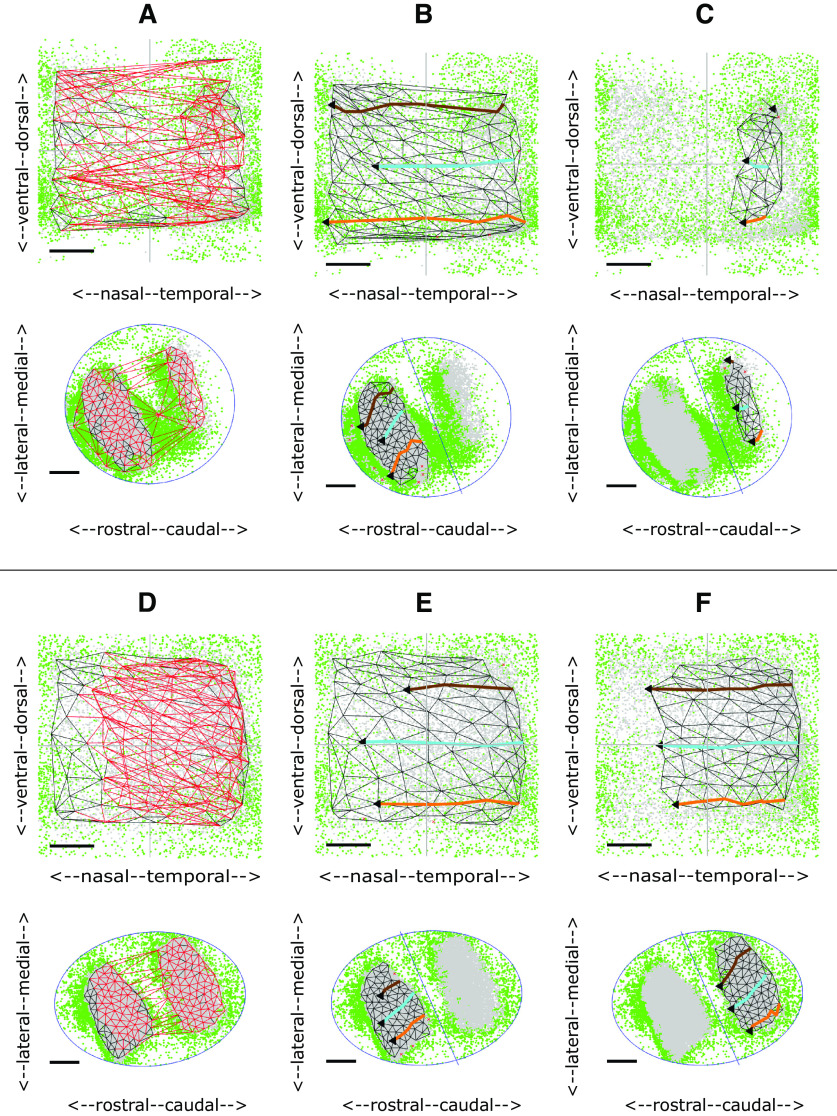
2D maps for two HOM datasets. ***A–C***, Maps formed from the data shown in Figure 2*I*. ***A***, Whole map with 148 nodes. Largest ordered submap has 123 nodes with Map Quality 83%. ***B***, ***C***, Subpartmaps, with Map Quality of 93%, 10% more than in the largest ordered submap. VFD: 17%. All polarities between 86% and 91%. AM, EM: 212°/mm, 85°/mm (***B***); 79°/mm, 85°/mm (***C***). ***D–F***, Maps formed from the data shown in Figure 2*J*. ***D***, Whole map with 177 nodes. Largest ordered submap has 131 nodes with Map Quality 74%. ***E***, ***F***, Subpartmaps, with Map Quality of 98%; 24% more than in the largest ordered submap. VFD: 63%. All polarities between 91% and 93%. AM, EM: 174°/mm, 117°/mm (***E***); 140°/mm, 85°/mm (***F***). Conventions as in Figure 3. Note that the homozygote map in ***A–C*** is based on the data presented by Owens et al. (2015) as the paradigm heterozygote with a double map (their Fig. 1*N*).

Figure 6*A–C* shows the HOM map with the smallest VFD, of 17%, with pixel distributions in Figure 2*I*. The projection from rostral colliculus covers most of the visual field (Fig. 6*B*) and the small projection from caudal colliculus (Fig. 6*C*) is to temporal field only. Figure 6*D–F* show the HOM map with the largest VFD, 59%, with pixel distributions in Figure 2*J*. Apart from far-nasal visual field, there is a double representation on the colliculus.

### Summary of 2D maps

Highly ordered 2D maps from colliculus to visual field were reconstructed from noisy Fourier imaging data derived from both WT and EphA3 knock-in mice. Sampling the colliculus at a spacing of 80 μm enables 2D maps to be constructed with around 90% of neighboring points in the correct relative order and orientation.

In a WT map there is a single well-ordered map and HETA maps are identical to WT maps. In HETBs, HETCs and HOMs, the colliculus can be divided into two areas to produce two ordered maps. Figure 9*A* justifies this division, showing that more nodes are used in the two largest ordered partmaps than in the single map. The VFD in the two partmaps varies both within and between groups. On average, it is greater in the HOMs, less in the HETCs, and very small in the HETBs (Fig. 9*B*). In HETCs and HOMs, the area of visual field that is represented in both areas is restricted to temporal field, nasal field being absent from caudal colliculus (Figs. 5, 6). Azimuthal magnification is significantly greater in the rostral map than in the caudal map, being greatest in the HOM maps and least in HETB maps (Fig. 9*E*,*F*). Figure 9*A–F* give summary statistics for all 21 cases.

The large degree of variability within both HET and HOM maps suggests that they are derived from a continuum of maps ranging from a single ordered map covering the colliculus to a map with a projection of visual field from two distinct areas on the colliculus. Here, more of the visual field is represented rostrally than caudally where the representation of nasal field tends to be absent.

Since these maps are from colliculus to visual field, they do not give information about the individual projections of the EphA3+ and EphA3− retinal cell populations. It seems reasonable to conclude that in groups HETA and HETB, the projections from the populations of EphA3+ and EphA3− cells overlap completely or almost completely to give a single projection with little or no overlap in visual field, yielding effectively single wild-type maps.

In double maps, one possibility is that the rostral projection is from EphA3+ cells exclusively and the caudal projection from EphA3− cells exclusively. However, this implies that a substantial part of the representation of EphA3− cells on the caudal projection area would be missing (Figs. 5*C*,*F*, 6*C*,*F*). It seems unlikely that these missing representations of nasal field are to the central part of the colliculus which cannot be mapped by Fourier imaging because of interference between two projections. This is because the two interfering projections would be from far-nasal and far-temporal field (Fig. 1*C*, green areas), whereas in the HETC and HOM maps temporal field is always represented. Another possible explanation is that in temporal retina (corresponding to nasal visual field) there are no functioning EphA3− cells.

### Insights from modeling studies

To explore these issues requires study of double maps in which the projections from retina to the colliculus can be related to the projections from colliculus to visual field (or retina) supplied by Fourier-based intrinsic imaging. This information is available in modeling studies and we looked at double maps obtained from the energy minimization model TK2011 (Triplett et al., 2011). To compare with the experimental maps, we made Lattice plots of the maps from colliculus to retina after applying our simulation of the Fourier imaging process to the model data. These maps were supplemented by Lattice plots from retina to colliculus, including separate maps of the EphA3+ and the EphA3− projections. Retina was plotted as visual field to ease comparison with the experimental data.

**Figure 7.**
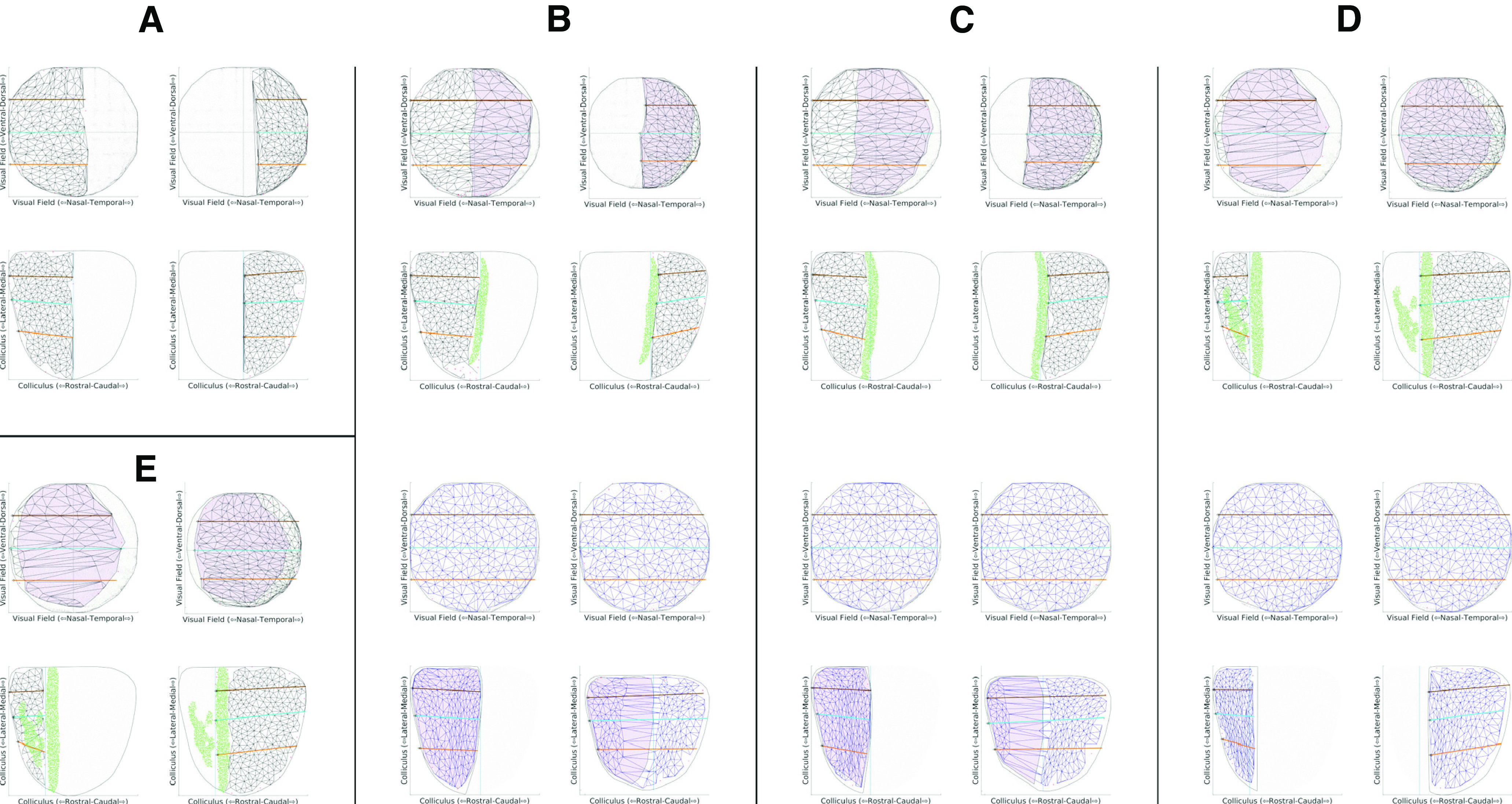
Single runs of the TK2011 model (Triplett et al., 2011). Connections developed between 10,000 retinal cells and 10,000 collicular cells, using the code developed by Hjorth et al. (2015). Maps were computed for different values of DR, the amount of EphA added to the Islet2 cells, and constructed using the Lattice Method (Willshaw et al., 2014). Largest ordered subpartmaps are shown. Retina is plotted as visual field to aid comparison with the experimental plots. For the mapping from colliculus to retina, colored black, the collicular lattice was divided into two by a line through the center of the green (rejected) pixels. The left-hand plot is the map from the rostral part of the colliculus; the right-hand plot that from the caudal part. For the mapping from retina to colliculus, colored blue, the left-hand plot is the projection from EphA3+ cells and the right-hand plot that for the EphA3− cells. Map orientation shown as in the experimental plots. ***A***, DR = 0. Colliculus to retina map only. The collicular lattice was divided into equal-sized rostral and caudal halves. ***B–D***, Projections in both directions are shown for DR = 0.22, 0.34, 0.56. For the projection from colliculus to retina the shaded region in the retina is represented in both rostral and caudal collicular areas. ***E***, DR = 1.12. Colliculus to retina projection only. Each retinal cell was chosen to be EphA3+ with probability 0.5. Parameter values: α = 90, β = 135, a = 0.03, b = 0.11 (Triplett et al., 2011; Hjorth et al., 2015); γ = 0.00625, 5 × 10^8^ iterations (Gale, 2022). The EphA gradient is derived from Reber et al. (2004). Values of DR quoted are expressed as a fraction of the span of the values of EphA over the WT retina. On this scale, the values of DR for an EphA3^ki/+^ and an EphA3^ki/ki^ map (Reber et al., 2004) are 0.37 and 0.74, respectively.

Figure 7 shows a sequence of maps for different values of DR, the extra amount of EphA added to EphA3+ cells. The values of the key measures are given in Table 1. With DR set at zero, in the collicular to retina projection, colored black, rostral colliculus maps to temporal retina (visual field) and caudal colliculus to nasal retina (temporal field); there is a single map (Fig. 7*A*). At a higher value of DR (Fig. 7*B*), there is an area on the colliculus colored green and rejected by filtering because the average retinal position varies considerably. This defines two distinct projection areas. The projection from rostral colliculus covers most of the retina while that from caudal colliculus is restricted to nasal retina, giving a measurable VFD. As DR is further increased, the projection to the retina from caudal colliculus increases (Fig. 7*C*,*D*), with larger VFD, until both rostral and caudal areas project to almost all of the retina (Fig. 7*E*). To show the variation in map properties over different runs of the model for a given DR and how these properties depend on DR, three statistics were examined: the VFD and the quality of the whole map and in the rostral and caudal partmaps. A total of 100 maps were generated at a resolution of 2000 nodes per map, the lower resolution needed to give manageable run times. Figure 9 shows the interquartile distributions of these statistics for a range of DR. For a given DR, the VFD (Fig. 9*G*) varies over a small range. As DR is varied, there is a critical point at approximately DR = 0.1 where irregularities in the single map emerge, followed by a smooth monotonic increase in mean VFD. The quality of the whole map (Fig. 9*H*) shows a steady decrease with increasing DR while the partmap qualities remain consistent except around the critical point. These statistics support the idea that, despite the stochasticity in the model, map formation given a particular DR value is relatively consistent and that there is no abrupt phase transition between single maps and double maps.

These simulated maps from colliculus to retina can be related directly to the experimental maps and have several similar features. Simulated WT maps are single. A simulated EphA3 knock-in map can be single, or a double map where the proportion of the retina (visual field) represented in both projection areas varies widely, depending on the value of the EphA3 augment, DR. In the double maps a larger extent of the retina is represented rostrally than caudally with much of temporal retina (nasal field) not represented in the caudal area. In the experimental maps there is significant variation, such as within the HOM category where either a small or a large extent of visual field is represented in rostral colliculus (Fig. 6*C*,*F*). This difference could be attributed to the fact that, unlike in the simulations, signal strength varies considerably over the colliculus (Kalatsky and Stryker, 2003; Cang et al., 2008). The smaller amount of colliculus available for constructing the map after filtering may also explain why in the simulations, ordered maps of 400–500 nodes were constructed compared with 150–200 for the experimental maps.

**Figure 8.**
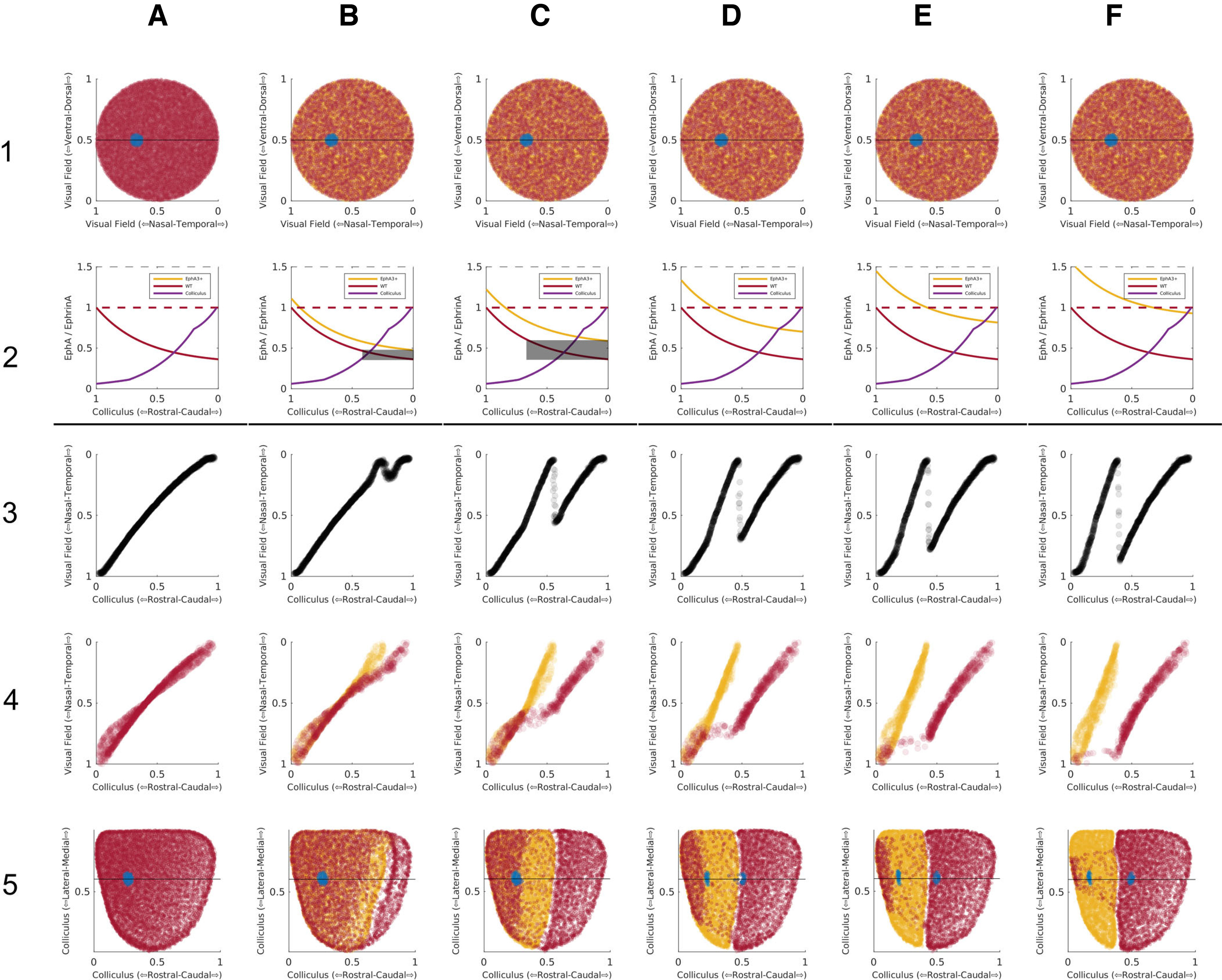
Data of the type used to generate Figure 7 from the TK2011 model was plotted to show a sequence of six 1D projections of the retina measured along a straight line located at dorsoventral position 0.5 and running nasotemporally onto the colliculus measured along the matching straight line running rostrocaudally, for increasing values of DR. Retina plotted as visual field. Each subfigure has five separate plots. Row 1, Distribution of EphA3− (purple) and EphA3+ (orange) cells over the retina. The horizontal line indicates the line along which the 1D profile was taken. Blue marks the site of a simulated injection of 1% of the retinal cells (row 1) and its projection(s) on the colliculus (row 5), which define(s) the mediolateral position of the matching line. Row 2, The distribution of EphA along the nasotemporal axis of the retina for both EphA3− cells (purple) and EphA3+ cells (orange) and of ephrinA cells (magenta) across the rostrocaudal axis of the colliculus. Nasal visual field corresponds to rostral colliculus and temporal field to caudal colliculus. Row 3, Nasotemporal component of the positions of all retinal cells with contacts from the cells lying along the rostrocaudal axis of the colliculus at mediolateral position 0.5, marked in row 5. Row 4, Rostrocaudal component of the positions of all collicular cells with contacts from the retinal cells lying along the nasotemporal axis of the retina at dorsoventral position 0.5, shown in row 5. The projections from the EphA3− cells (purple) and the EphA3+ cells (orange) can be distinguished. Row 5, Distribution of the average positions on the colliculus of the projections from each retinal cell, with the projections from the injected cells in blue. Initially a large number of contacts are made at random and then contacts are withdrawn selectively, those that lower the overall energy being maintained. The difference in energy between the contacts made by two retinal cells on the same collicular cell is the product of the amounts of EphA and ephrinA present. The contact with more EphA3 tends to be withdrawn as this decreases the energy by a larger amount. Note that, as in all Markov Chain Monte Carlo methods (Brooks et al., 2011), this is not a true minimization model which converges to a single configuration, but rather to a distribution of possible configurations (Gale, 2022). ***A***, DR = 0.0. EphA3− and EphA3+ cells at the same retinal location have the same amount of EphA and so there is a single map (Fig. 7*A*). ***B***, DR = 0.16. Some EphA3− cells from nasal retina (temporal visual field) have values of EphA lower than those in the EphA3+ population at the same collicular position (row 2, gray bar) and so survive at the expense of the contacts from the EphA3+ cells there. The contacts made more centrally by these same EphA3+ cells now compete against the EphA3− cells there, which have higher energy. The EphA3+ cells tend to survive, and the overall effect is that the EphA3+ projection from nasal retina moves more centrally. ***C***, DR = 0.32 (Fig. 7*B*). ***D***, DR = 0.48 (Fig. 7*C*). There is a growing population of EphA3− cells with unique values of EphA, increasing the extent of the single EphA3− projection caudally and leading to contacts from more and more EphA3+ cells being removed from this region of the colliculus. There is still a double projection far rostrally, where the activity mechanism favoring neighbor-neighbor connections dominates over the weaker chemoaffinity mechanism. ***E***, DR = 0.64. ***F***, DR = 0.78 (Fig. 7*D*). As DR is increased, the effect of chemoaffinity rostrally becomes stronger, diminishing the superposed projection. For sufficiently large DR, all the EphA3− cells have values of EphA lower than the EphA3+ cells (row 2) and a double projection results. EphA3+ cells project rostrally and EphA3− cells caudally.

Examination of the simulated projection from retina to colliculus now enables identification of the separate projections from the EphA3+ and the EphA3− cells. As described above, for DR = 0, both EphA3+ and EphA3− cells from all parts of the retina project across the entire colliculus to give a single map. For a larger value of DR (Fig. 7*B*, blue maps), the projection from EphA3+ cells becomes restricted to rostral colliculus and EphA3− cells project more widely so that rostral colliculus is innervated by both EphA3+ and EphA3− cells. At a larger value, the projection from EphA3− cells starts to become restricted to caudal colliculus with a strip of double innervation rostrally (Fig. 7*C*), which becomes smaller for larger DR (Fig. 7*D*). Ultimately EphA3+ cells project rostrally and EphA3− caudally to give a double representation of retina on the colliculus. Figure 8 shows a set of 1D profiles for a series of maps where the value of DR was increased systematically in steps of 0.16 from 0.0 to 0.64. This illustrates how connections are modified during development, as described in the legend. Rows 3 and 4 in Figure 8 illustrate the difference between a 1D map of the retina (plotted as visual field) seen from the colliculus and that of colliculus seen from the retina.

In summary, according to this model, connections are formed under the three influences of chemoaffinity, neural activity and competition. The chemoaffinity mechanism is of the graded matching type (Prestige and Willshaw, 1975) and the level of neural activity controls the tendency of neighboring retinal cells to connect to neighboring collicular cells.

There are two other relevant properties of the model (our unpublished data).

1. Reducing the level of neural activity slows down the development of the projection in its earliest stages. This also reduces the number of EphA3+ and EphA3− cells from the same retinal location projecting in register on rostral colliculus.
2. In double maps the distribution and relative proportion of EphA3+ to EphA3− cells in the retina affect their patterns of innervation. For example, changing the percentage of cells that are EphA3+ changes the proportions of the colliculus innervated by the two populations.

In these simulations it is how the profile of EphA over the EphA3− cells compares with that over the EphA3+ cells which is crucial in determining the form of an EphA3 knock-in map. As seen by Fourier-based imaging, if the two profiles are almost identical, a single or near single map would result [HETA (Fig. 3); HETB (Figs. 4, 8*B*)]. If the two profiles are displaced, a partially double map results [HETC (Fig. 5); HOM (Fig. 6), (Fig. 8*C–F*)]. The degree of displacement determines the form of the double map.

We repeated the simulations of the computational model which Owens et al. (2015) used to support their stochastic hypothesis. This model, TK2006 (Tsigankov and Koulakov, 2006, 2010) is an earlier version of the one that we used in our analysis of the experimental data. We used this model to give a direct comparison; we did not use it in our analysis of the experimental data as it allows only one contact per cell, which disrupts the mapping, as described below.

**Figure 9.**
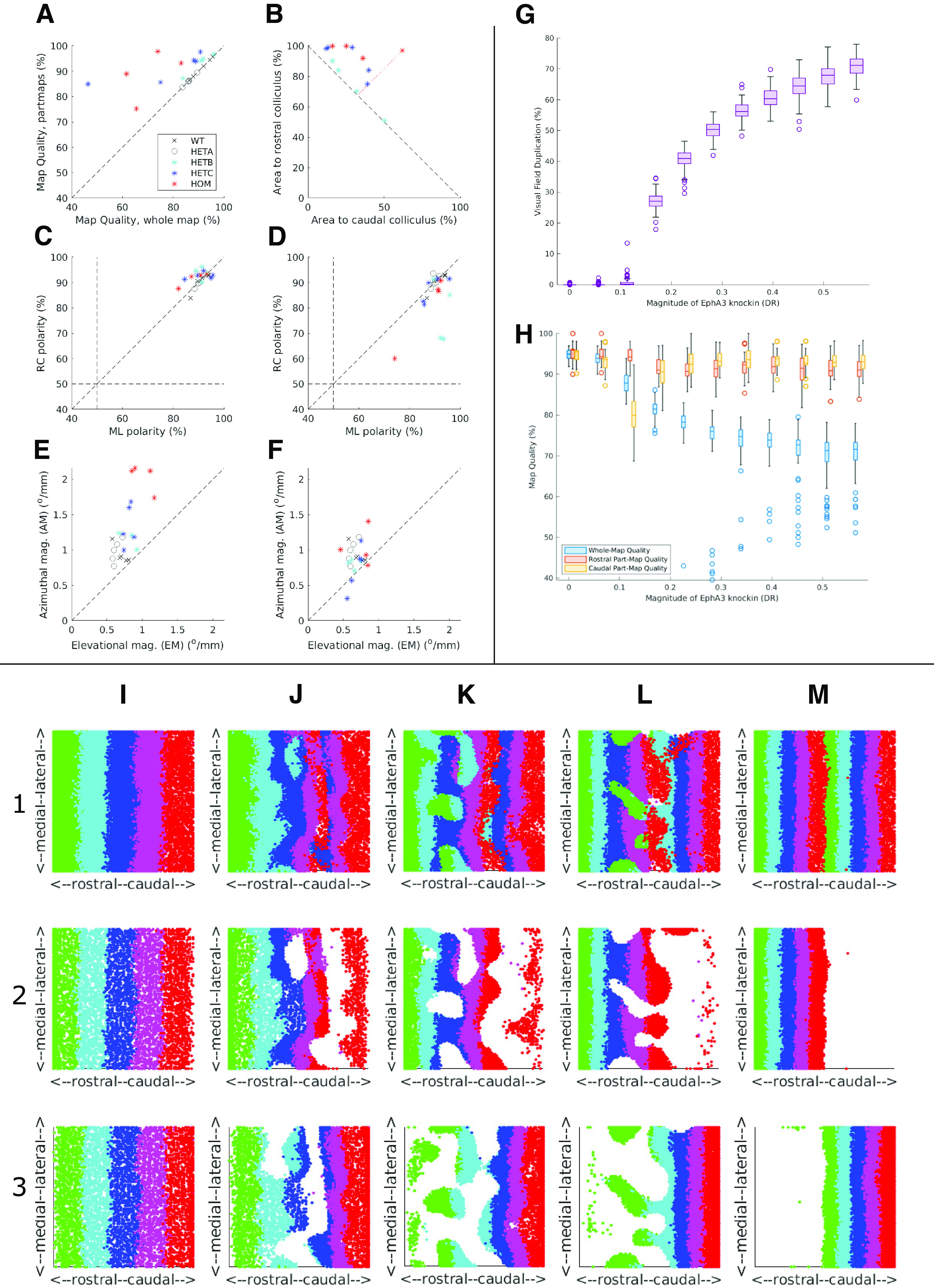
Summary and controls for both experimental and modeling data. ***A–F***, Summary data for the 21 experimental maps analyzed. ***A***, Map Quality of the largest ordered two partmaps plotted against Map Quality for the largest ordered submap. For the single maps identified in the WT and HETA categories, the same Map Quality is plotted on both axes. Data points above the diagonal indicate that the partmaps cover more of the colliculus than the whole map. ***B***, For the HETs and the HOMs where two projection areas were identified, the relation between the area of visual field represented in the rostral area compared with that in the caudal area. The VFD is equal to the perpendicular distance from the given point to the off-diagonal. ***C***, ***D***, Rostrocaudal (RC) polarity plotted against mediolateral (ML) polarity in double maps for the rostral map (***C***) and the caudal map (***D***). ***E***, ***F***, Azimuthal magnification (AM) plotted against elevational magnification (EM) in double maps for the rostral map (***E***) and the caudal map (***F***). In ***C–F***, data from the single maps is included. ***G***, ***H***, Summary data for multiple simulations conducted on the TK2011 model of reduced size for different amounts of the EphA3 knock-in, DR. ***G***, VFD. ***H***, Map Quality for both largest ordered submaps and partmaps. 2000 retinal cells and 2000 collicular cells, 2 × 10^7^ iterations per run. 100 runs for each value of DR. Mean shown by the horizontal line within the box, which encloses one quarter of the data points. Standard deviation shown by the vertical line. ***I–M***, Maps generated by simulations of the TK2006 model (Tsigankov and Koulakov, 2006) for five different values of the EphA3 augment, DR, to be compared with those in Figure 7 of Owens et al. (2015), using the same code (M. Reber, personal communication). The retinal cells are labeled according to their position along the nasotemporal axis by five stripes of different colors (Owens et al., 2015). For each value of DR, there are three maps showing the color-coded nasotemporal origins of the retinal cells (plotted as visual field) projecting to each part of the colliculus. Row 1, Projection from all cells. Row 2, Projection from EphA3+ cells. Row 3, Projection from EphA3− cells. ***I***, DR = 0.3 (0.35). ***J***, DR = 0.36 (0.42). ***K***, DR = 0.4 (0.46). ***L***, DR = 0.5 (0.58). ***M***, DR = 0.7 (0.81). In the TK2006 and TK2011 models, the values of DR were normalized to different bases. To aid comparison with the simulations shown in Figures 7 and 8, the corresponding values for TK2011 are shown in parentheses.

We used the same set of parameters as Owens et al. (2015) and varied the magnitude of the EphA3 augment, DR, over the same range. The maps in the first row of panels of Figure 9*I–M* resemble their published maps. At low DR, there are single maps (Fig. 9*I*) and at higher values completely double maps (Fig. 9*M*). With a change of DR from 0.3 (Fig. 9*I*) to 0.36 (Fig. 9*J*), a single map became a blotchy map of the type that Owens et al. (2015) found, which transformed gradually into a double map over the range DR = 0.36 to DR = 0.7 (Fig. 9*J–M*). In contrast, Owens et al. (2015) reported that a slight variation of DR around 0.36 would switch the map from single to double. Plotting the projections from the EphA3+ and EphA3− retinal cells separately (rows 2 and 3; Fig. 9*I–M*) revealed that the blotchy maps are due to neighboring cells of the two types forming interleaving projections, each excluding the contacts from cells of the other type, similar to a pattern of zebra stripes This is a result of the strong competitive constraint of requiring one-to-one connectivity which masks the smooth variation from a single projection to a double projection as DR is increased.

In summary, our simulations with this model show that, as DR is increased from 0.3 to 0.7, the map evolves gradually from a single into a double map, by DR = 0.7. We did not find any evidence for small variations of DR producing rapid switching between the single and double states.

## Discussion

In the original analysis of this dataset, Owens et al. (2015) sampled data from the azimuthal scan along three fixed rostrocaudally orientated tracks to produce 1D maps, each classified as single or double using their quantitative measure. In different EphA3^ki/+^ mice, at the three locations sampled they found single maps (as in WTs), double maps (as in EphA3^ki/ki^ mice) or a mixture of the two at different positions along the mediolateral axis (Fig. 1*B–D*). Eliminating both genetic variation and variation in neural activity, they concluded that the mixed category of map represents an unstable transitional state between those of a single and a double map, which can be interchanged by small fluctuations in the competing forces of chemoaffinity and neural activity acting on the innervating nerve fibers.

Our 2D maps were constructed from data from both elevation and azimuthal scans using the Lattice Method (Willshaw et al., 2014), a general method for constructing topographic maps. It provides quantitative measures of local and global ordering which can be used to compare maps without assuming a particular map scale or orientation. Statistics derived from this method can be used in population studies generated from experiment or from simulation (Fig. 9*G*,*H*; Gale, 2022). Sampling the colliculus at a resolution of 80 μm enables 2D maps to be constructed in which over 90% of neighboring points are in the correct relevant order and orientation. With standard intrinsic imaging the resolution was 120 μm (Mrsic-Flogel et al., 2005) and for extracellular recording 200 μm (Dräger and Hubel, 1976). The Lattice Method has wide applicability and could be applied to other datasets such as phase maps derived from ultrasound data in mouse (Macé et al., 2018) and using Calcium imaging in *Xenopus* (Li et al., 2022).

In EphA3^ki/+^ and EphA3^ki/ki^ mice we found a variety of maps, ranging from an entirely single projection to a double map with a rostral area on the colliculus receiving a projection from a large extent of the visual field and a caudal area receiving a smaller projection, consistently from temporal visual field, part of the visual field being represented twice.

Without matching azimuthal and elevational scans, we could not construct 2D maps for the small number of cases relating to the roles of genetic diversity and neural activity that Owens et al. (2015) analyzed. Their interpretations rely on their method of classifying 1D maps as single, double or mixed, which lacks validation using ideal data. Small changes in the decision boundaries in their 2D representation of their data would lead to different classifications; see, for example, their Figures 2D and 6F.

The main difference between our approach and that of Owens et al. (2015) is that we constructed full 2D maps using filtered data from both azimuthal and elevational scans rather than sampling the 1D projection from unfiltered data from the azimuthal scan. Filtering is needed to remove the ambiguous reports from parts of the colliculus receiving projections from different parts of the visual field.

Owens et al. (2015) also presented results from the computational model TK2006 which they argued showed the type of stochasticity that they advocated. When we reran their computer simulations using the same parameter values, we found that the one-to-one mechanism of competition introduced artefacts.

TK2011 is a revised version of this model allowing for multiple contacts between cells (Triplett et al., 2011; Hjorth et al., 2015), which we analyzed in detail. It fits the experimental data qualitatively. In this model, in some cases the projections from EphA3+ and EphA3− retinal ganglion cells are superposed in rostral colliculus. This is one property of a graded matching (Prestige and Willshaw, 1975) chemoaffinity model where in rostral colliculus there is a relatively small difference between the chemoaffinity signal carried by the two classes of innervating retinal fibers (Reber et al., 2004). TK2011 is not unique, with the disadvantage that the notion of energy minimization is difficult to translate into the biology. Adding a fiber-fiber interaction term to the energy function reflecting the chemical signals from retinal fibers innervating the same collicular cell (Gebhardt et al., 2012) introduces unnecessary model complexity.

This model is consistent with findings that knock-in of the EphA3 receptor combined with knock-in of ephrinA3 (Savier et al., 2017) reversed the effect of EphA3, producing single maps. One implication is that each ephrinA may target only its matching EphA. If so, this would require a re-evaluation of current models. For example, retinal ephrinA5 would then mask retinal EphA5, assumed in most models to contribute to chemical signaling. The model is also consistent with ephrinA5 knock-out results where conditional retinal knock-out had no effect and conditional collicular knock-out degraded the map (Suetterlin and Drescher, 2014).

In the maps in EphA3 knock-in animals subject to knock-out of the β2 receptor used by Owens et al. (2015) to explore the role of neural activity, it is unclear as to how the effects of β2 knock-out should be modelled (Stafford et al., 2009). We found that lowering the strength of activity in the model reduces the extent to which EphA3+ and EphA3− cells project in register on rostral colliculus.

The consistent absence in our double maps of caudal colliculus receiving a projection from nasal field could be caused by EphA3− cells and EphA3+ cells of temporal origin projecting in register to rostral colliculus, in line with axonal tracing (Brown et al., 2000; Reber et al., 2004; Bevins et al., 2011; Owens et al., 2015) and as found in the modeling. This effect could be investigated in each case by looking for a coincidence of EphA3− and EphA3+ fibers projecting to the appropriate part of the colliculus. For example, in the case shown in Figure 6*A–C*, there should be a large, superposed projection in medial colliculus; for Figure 6*D–F*, there should be little or none. If there is no superposed projection, the missing part-projection, presumably from the EphA3− cells, must be made elsewhere. Alternatively, there could be an inhomogeneous retinal distribution of the Islet2+ cells to which EphA3 is attached. Pak et al. (2004) found a weaker representation of Islet2+ cells in the ventrotemporal crescent of the retina than elsewhere in the retina. Marcucci et al. (2019) found that neurogenesis of retinal ganglion cells in ventrotemporal retina lags behind that in dorsonasal retina.

### Alternative hypotheses

The hypotheses compatible with the original 1D analysis are: (1) in EphA3^ki/+^ maps there is stochasticity in the mechanisms making the connections on the colliculus (Owens et al., 2015); (2) the level of EphA3 in Islet2 cells varies systematically over the retina. Our 2D maps present a different picture of the data from the original 1D analysis which seems to rule out both hypotheses. In the EphA3 knock-ins we found a variety of double maps from both EphA3^ki/ki^ and EphA3^ki/+^ animals where the variation is along the rostrocaudal axis, which concords with the anatomic evidence (Brown et al., 2000); we did not find any mixture of single and double maps at different locations along the mediolateral axis.

The substantial variability in the extent of the double projections from animal to animal occurring in both genotypes suggests another hypothesis: (3) the relative amount of the induced EphA3 receptor compared with the levels of endogenous retinal EphA varies between animals. A similar systematic variability is seen in the computer simulations as the level of EphA3 is increased from zero (Figs. 7, 8, 9*G*). Several authors have quantified the amount of knocked-in EphA3 in Islet2 retinal ganglion cells. Brown et al. (2000) used *in situ* hybridization and presented their data schematically. From mRNA measurements they inferred that the level of EphA3 in the homozygous knock-in is twice that of the heterozygous knock-in although no quantitative measurements were given. Reber et al. (2004) showed that the levels of EphA3, EphA4, EphA5 and EphA6 in the retina have a standard deviation of around 10% for each receptor type. This could be sufficient to account for the variation in the maps given that it is the relative levels of EphA3 which is important.

To explore the third hypothesis requires examining how the two populations of EphA3− and EphA3+ retinal cells project to the colliculus and relating the form of each functional EphA3 knock-in 2D map to (1) the distribution of Islet2 cells over the retina, the density of EphA3 in different heterozygotes and (2) the location of collicular cells which are innervated by both EphA3+ and EphA3− cells. Substantial variation between the 2D maps on the two colliculi would rule against this hypothesis. Fourier-based imaging has the advantage of providing data from over the entire brain structure assessed but it requires surgical intervention, making it difficult to combine with methods targeting one class of retinal ganglion cells. Functional ultrasound may be more suitable, being applied externally (Macé et al., 2018). Combining functional ultrasound with optogenetics (Brunner et al., 2020) to look at the EphA3+ projection from the Islet2 cells alone may be possible to avoid the problem of ambiguous responses from part of the colliculus holding projections from widely different parts of the visual field.

**Table 1:** Summary data for the single runs of model TK2011 (Figure 7)

Figure	A	B	C	D	E
DR	0.0	0.22	0.34	0.56	1.12
Number of nodes	485	436	415	426	407
VFD	1	47	58	74	76
Map Quality (largest ordered submap)	97	80	74	54	73
Map Quality (largest ordered partmaps)	98	95	96	95	95

## Synthesis

Reviewing Editor: Lynda Erskine, University of Aberdeen Decisions are customarily a result of the Reviewing Editor and the peer reviewers coming together and discussing their recommendations until a consensus is reached. When revisions are invited, a fact-based synthesis statement explaining their decision and outlining what is needed to prepare a revision will be listed below. The following reviewer(s) agreed to reveal their identity: Michael Reber. Note: If this manuscript was transferred from JNeurosci and a decision was made to accept the manuscript without peer review, a brief statement to this effect will instead be what is listed below.

Apologies again for the delay in reviewing your manuscript. Appended below are the reviews. Both reviewers found this to be a very well conducted and interpreted study, but have a few suggestions for changes to the manuscript prior to publication. I have not prepared a single synthesis of the reviews as I thought it would be useful for you to see the reviewers’ comments in their entirety. Thank you for submitting your interesting work to eNeuro. I look forward to receiving your revised manuscript.

This manuscript presents a great piece of computational neuroscience work which re-addresses a longstanding debate in visual map formation. Using the lattice method, it explores with great details the singularity of visual mapping in one particular set of models, the Isl2-EphA3KI animals, generated and first studied 20+ years ago. Previous anatomical and functional analyses in this animal model, especially in the heterozygous Isl2-EphA3KI/+, revealed unique but complex mapping patterns which have been challenging to explain and understand. This work is providing new insights into principles of visual map organization.

I would suggest the authors to expand their discussion and consider, in their alternative hypotheses section, recent molecular mechanisms such as fiber-fiber interaction (Suetterlin and Drescher, 2014) and how this could affect the outcome of their model, in particular in Isl2-EphA3KI/+, where DR between 0.3 and 0.7 show gradual map duplication.

Moreover, a recent anatomical analysis showed that inactivation of ectopic EphA3 expression in the Isl2-EphA3KI/+ animals by co-expressed ephrin-A3 ligand, restored single retino-collicular map (Savier et al., 2017). How could this mechanisms be further considered in the model presented here?

Haploinsufficiency as a possible explanation for EphA3 variation in the retina is very unlikely as such process would created large variation of EphA3 level between neighboring RGCs which would not generate the gradual transition to duplicated maps as proposed by the model. Instead, the 10% standard deviation of the summed EphA receptors expression level revealed in Reber et al., 2004, and mentioned here would suffice to explain the transition from single to double maps for DR within 0.36 to 0.7.

Minor concern:

The labelling of the dorsal-ventral visual field axis in Figures 7 and 8 (row 1) is opposite compared to all other figures. As a consequence, the color-coded elevation lines (brown being the dorsal-most and orange the ventral-most) do not correspond (brown now being ventral-most) and confuses the reader.

By conducting a novel analysis of the retinocollicular map in Islet2-EphA3 knock-in mice, this study demonstrates that maps are much more diverse than previously thought. This diversity puts into question previous models of map formation and suggests that other mechanisms underlie the formation of visual maps.

This manuscript reports a novel analysis and careful examination of the retinocollicular map of homozygous and heterozygous Islet2-EphA3 knock-in mice after reconstructing 2D visuocollicular maps with the Lattice Method. Results from this study demonstrate that the double maps observed in both heterozygotes and homozygotes are diverse and suggest that mechanisms distinct from a stochastic process at the colliculus are responsible for the variability observed. Overall, this complex modeling study is thoroughly conducted and very well explained. It should be of high interest to readers interested in the formation of neural maps and is thus appropriate for publication in eNeuro, provided the comments below are addressed.

1) The authors suggest that different levels of EphA3 across EphA3ki/+ animals might explain the variability of visuocollicular maps observed. However, Owens et al., 2015 observed different maps in the right and left colliculi of individual mice. The authors should discuss how their model would explain such results.

2) Owens et al., 2015 reported that EphA3ki/+ animals no longer have double maps in a β2−/− background, which indicates a role for neuronal activity in the variability of visuocollicular maps. The author should discuss further this result in the context of their new analysis and model.

3) In the discussion, it is not clear how haploinsufficiency would apply to a knock-in model and explain different levels of EphA3 in EphA3ki/+ animals.

4) While the manuscript is very well and clearly written, it might be challenging to read for readers who do not have any background in retinotopic mapping. Expanding the introduction to describe in more detail the map itself and the expression of EphAs and ephrins in the retina and colliculus in WT might make the manuscript more accessible.

Minor points:

5) Figures G and H are not described in the main text.

6) Please indicate the DR values in Figure 8.

7) In paragraph 2 of introduction, “expressed by Islet class of retinal ganglion cells” might be more appropriate than “bound to”.

8) EphA3 is usually described as a receptor rather than a ligand.
